# Development and validation of the Women’s Self-care Knowledge and Attitude Questionnaire (WSKAQ)

**DOI:** 10.1186/s12889-024-19831-w

**Published:** 2024-08-28

**Authors:** Khadijeh Khademi, Mohammad Hossein Kaveh, Abdolrahim Asadollahi, Mahin Nazari

**Affiliations:** 1 PhD candidate of Health Promotion, Student Research Committee, Department of Health Promotion, School of Health, Shiraz University of Medical Sciences, Shiraz, Iran; 2 Research Center for Health Sciences, Institute of Health, Department of Health Promotion, School of Health, Shiraz University of Medical Sciences, Shiraz, Iran; 3 Department of Health Promotion and Aging, School of Health, Shiraz University of Medical Sciences, Shiraz, Iran; 4 Department of Health Promotion, School of Health, Shiraz University of Medical Sciences, Shiraz, Iran

**Keywords:** Self Care, Knowledge, Attitude, Validation, Menopause

## Abstract

**Background:**

Menopausal women should learn self-care practices to enjoy a healthy lifestyle, positive health behaviors, and health status. In addition, the lack of self-care knowledge can lead to unhealthy attitudes and lifestyles, resulting in many complications. Improved self-care knowledge can foster positive attitudes, leading to healthier lifestyles as a beneficial cycle. However, menopausal women have limited knowledge about self-care and evaluation tools. Therefore, this study aimed to develop and investigate the validity and reliability of the Women’s Self-care Knowledge and Attitude Questionnaire (WSKAQ) in Iranian menopausal women.

**Methods:**

This cross-sectional study was conducted on 249 menopausal women, aged between 45 and 65 years and at least one year post-menopause, with a minimum literacy level of elementary education. Validation properties included construct validity, exploratory (EFA), and discriminant validity. Reliability was further established through Cronbach’s α and McDonald’s Omega. PASS 15 and SPSS 27 software were used to select centers, calculate sample size and analyze the data, respectively.

**Results:**

The initial stage of construct validity involved the Kolmogorov–Smirnov test and EFA, resulting in a 6-item self-care knowledge subscale, 47.29% of the total data variance, and 7-item self-care attitude subscale, 55.50% of the total data variance. Independent t-test indicated that menopausal women with education level equal to or higher than diploma have significantly higher self-care attitude scores than those with lower than diploma (*p* = 0.007). Additionally, significant correlations were observed between self-care knowledge and attitude (r:0.30). Cronbach’s α and McDonald’s Omega coefficients of the 13-item WSKAQ were 0.77 and 0.78, respectively.

**Conclusions:**

Based on the results, the WSKAQ, consisting of 13 items, was validated and reliable for assessing the self-care knowledge and attitude of Iranian menopausal women.

## Introduction

Menopause is a normal stage in a woman’s life marked by the cessation of menstruation after 12 months of amenorrhea and its symptoms, which affect the health and quality of women’s lives [1, 2]. Menopausal women should learn self-care practices to enjoy a healthy lifestyle, positive health behaviors, and health status [3, 4]. Despite the importance of self-care to menopausal women’s health and quality of life, studies on menopausal women have reported their limited knowledge and activities about self-care. For example, in a study, only 0.8% of Iranian menopausal women performed appropriate self-care activities [5].

Self-care is a universally accepted concept [6]. Based on Orem’s nursing theory, self-care engages people to maintain, restore, or improve their health as strong, reliable, responsible, and capable of decision-making to care for their health appropriately [7]. The most recent state-of-the-science papers on Self Care list the following health behaviors as components of Self Care: (a) a healthy diet (with emphasis on water and fruit/vegetable consumption); (b) regular physical activity; (c) stopping smoking or tobacco use and alcohol intake; (d) obtaining regular preventive care (e) controlling anxiety or stress [8–11]. Lifestyle behaviors associated with self-care are influenced by race and ethnicity, and self-care should be understood within the context of a specific culture [12, 13]. In addition, the relationship between self-care knowledge and health is well-established [11, 14].

Among menopausal women, the lack of knowledge about self-care and unhealthy lifestyles are the cause of many severe complications in this period, which makes it necessary to improve self-care information [4]. For example, a study in Iran found that 45% of menopausal women had limited knowledge about self-care [5]. Enhancing awareness, knowledge, and understanding of self-care compensates, guides, enhances, and supports the self-care process, health condition, and quality of life [15–17]. Research on self-care knowledge, attitudes, and tools in menopausal women is limited. Existing studies tend to focus on self-care behavior in specific health conditions like chronic diseases, asthma, diabetes, and heart failure rather than examining self-care aspects among women overall, particularly menopausal women. Understanding knowledge and attitudes is vital as they impact behavior. In essence, most research has prioritized self-care behavior in specific diseases with minimal exploration of knowledge and attitudes towards self-care, especially among menopausal women [18–21].

Therefore, further research is needed to assess the quality of tools to assist researchers, policymakers, and clinicians in assessing self-care concepts to promote tailored care for specific group populations with different care needs [22]. The main limitation of long questionnaires is the vagueness of their validity and reliability. In addition, the brevity of the questionnaire is highlighted as a beneficial attribute for research purposes, ensuring quicker and more precise completion [23, 24].

The relationship between self-care knowledge, attitude, and health is apparent [11, 14]. Studies examining women’s self-care knowledge and attitude, specifically for menopausal women, appear to be lacking. The main objective of this study was to develop and evaluate the validation properties of the Women’s Self-care Knowledge and Attitude Questionnaire (WSKAQ) in Iranian menopausal women. The current study tried to develop a short questionnaire suitable for the menopause community, considering objective questions and covering different self-care knowledge and attitudes.

## Materials and methods

### Study design and population

This cross-sectional study was conducted from October to November 2023 on 249 menopausal women selected through convenience sampling from two urban health service centers in Shiraz, Iran, and randomly chosen using PASS software version 15 (NCSS, LLC., USA) [25].

The sample size was determined based on the mean ± SD of the self-care score (61.31 ± 22.35) from a similar study [26]. As a result of PASS 15 software (NCSS, LLC., USA) [25], 205 participants were calculated as the sample size for a type I error rate of 0.05%, a test power of 95%, and a 20% attrition rate. Finally, 249 menopausal women were included in the study.

Eligible menopausal women, aged between 45 and 65 years and at least one year post-menopause, with a minimum literacy level of elementary education, were invited to participate. The samples were required to sign an informed consent form to be included in the study. The exclusion criteria included reluctance to participate and failure to answer more than 20% of the questions in the questionnaire.

### Instruments

The study required participants to complete two questionnaires: the initial researcher-made questionnaire, the Women’s Self-care Knowledge and Attitude Questionnaire (WSKAQ), and a demographic information questionnaire.

The initial WSKAQ was a self-reported questionnaire containing 14 items; knowledge items (seven items) were scored on a scale of 0 for ‘false’ - ‘no idea’ and 1 for ‘true,’ and attitude items (seven items) based on five-point Likert scale ranging from 1 (strongly disagree), 2 (disagree), 3 (no idea), 4 (agree), to 5 (strongly agree), respectively. The questionnaire scores ranged from seven to 42, with higher scores indicating having better self-care knowledge and attitude.

The collected demographic data included age, menopause age (age at time that there was 12 consecutive months without experience of menstrual periods), education, marital status, occupation, and adequacy of family income for living expenses.

### Procedure

**Fig. 1:**
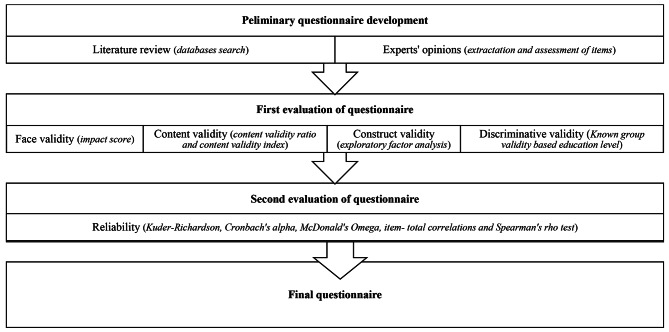
The stages of development and validation of 13- items WSKAQ

The initial questionnaire was developed based on an extensive literature review and experts’ opinions. Literature was searched on different databases, including Scopus, PubMed, Web of Science, and Google Scholar, with the following keywords: self-care knowledge, self-care attitude, women’s health, menopause, and post menopause. The search was not limited to a specific time interval. The research team extracted and then assessed the items used to evaluate self-care knowledge and attitudes among women in different studies. The following definition of self-care in scientific papers was used for developing the WSKAQ items: Self Care composite of emphasis on water and fruit/vegetable consumption, regular physical activity, stopping smoking or tobacco use and alcohol intake, obtaining regular preventive care such as screenings, and controlling stress [8–11]. In Islamic countries like Iran, smoking tobacco (0.77%), hookah smoking (3.64%), and alcohol consumption (1.35%) among women is even lower than it is in other countries around the world. Women in Iran are more likely to be socially active and less exposed to people with high-risk behaviors such as narcotic drug abuse or alcohol misuse [27, 28], and any item related to knowledge or attitude about smoking or tobacco use and alcohol intake was not considered. This study evaluated the WSKAQ psychometric properties to examine its content and construct validity and reliability. The stages of development and validation of WSKAQ are shown in Fig. 1.

### Face validity

Qualitative and quantitative face validity were evaluated to determine the face validity of WSKAQ. Ten menopausal women selected by convenience sampling were interviewed to evaluate the items’ difficulty, relevancy, and ambiguity. Then, the research team assessed the interviews and all comments implemented to edit statements. The impact score formula (Frequency (%) × Importance) was used to evaluate quantitative face validity. Items with an impact score ≥ 1.5 were considered appropriate for more analysis [29]. Then, the research team assessed the interviews and all comments implemented to edit statements.

### Content validity

The qualitative content validity was used to assess the statements based on grammar, wording, item allocation, scaling, clarity, and simplicity. Ten health education specialists familiar with the subject matter were asked to provide feedback to edit and revise the statements.

Quantitative content analysis was applied using the content validity ratio (CVR) and content validity index (CVI). CVR and CVI indices assess the necessity and relevancy of the items, respectively. A total of 16 specialists in health education, nursing, medical education, midwifery, and gerontology were asked independently to determine CVR and rate items using a three-point ranking scale (necessary, helpful but unnecessary, and unnecessary). Then, the CVR index was estimated using this formula: CVR=(nE-N/2)/(N/2), where nE signifies the number of panelists indicating “essential” and N donates the total number of panelists. Based on the Lawshe table, the minimum agreed CVR value evaluated by the 16 experts should be greater than 0.46 [30]. Finally, the mean CVR value of all self-care knowledge and attitude items was 0.80 and 0.81, respectively. Table 1 presents the CVR value of each questionnaire item, which is higher than the minimum acceptable range.

**Table 1:** The items of the 14- items WSKAQ for menopausal women in the Iranian women and their CVR, CVI

7-Items of self- care knowledge: Total CVR: 0.80; S-CVI^1^: 0.91.		CVR^2^	I-CVI^3^
**I1**	You should drink eight glasses of water daily.	0.50	0.93
**I2**	A healthy daily diet contains at least five units of fruits and vegetables.	1	0.89
**I3**	You should do 30 min of physical activity daily with increased heart rate and breathing.	1	0.91
**I4**	Every three years, women should take a cervix sample (Pap smear test).	0.87	0.93
**I5**	Women should perform breast self-exams every month.	0.87	1
**I6**	Every year, women should see a gynecologist/midwife for examination.	0.62	0.97
**I7**	Social support means getting help from others to solve life’s problems and issues.	0.75	0.77
**7-Items of self- care attitude**: Total CVR: 0.81; S-CVI: 0.86.			
**I1**	I believe that to achieve better health, I need to change my diet.	0.75	0.85
**I2**	I believe that consuming more fruits and vegetables plays a big role in preventing diseases.	0.75	0.80
**I3**	Regular and sufficient physical activity is necessary to maintain my health.	0.87	0.95
**I4**	Controlling my weight is essential to maintaining my health and preventing illness.	0.87	0.81
**I5**	Regular and periodic examinations and women’s health screenings reduce my concern about the diagnosis of advanced women’s cancers.	0.75	0.87
**I6**	My stress and mental tensions are reduced by asking for support and help from others.	0.87	0.91
**I7**	We can help each other perform self-care behaviors such as healthy eating and exercise by forming a self-help group with peers.	0.87	0.89

Note. *N* = 249. ^1^S-CVI: scale- content validity index, ^2^CVR: content validity ratio, ^3^I-CVI: item-content validity index

The CVI score of each item was calculated using Waltz and Bausell’s method [31]. Therefore, experts were asked to determine each item’s degree of relevance, clarity, and simplicity using a four-part spectrum. Then, the number of experts who chose options three and four was divided by the total number of experts. An I-CVI score over 0.75 was considered acceptable [31]. Lastly, the total CVI of self-care knowledge and attitude was calculated using the mean CVI scores for the entire item (Table 2) (0.91 and 0.86, respectively).

**Table 2:** Demographic characteristics of the menopausal women

Characteristics	*N*	%
**Marital Status**		
Married Widow Separated/ Divorced/ Single	196 36 17	78.7 14.5 6.8
**Education**		
Lower than Diploma Equal to or higher than Diploma	125 124	50.2 49.8
**Job**		
Homemaker Employed Retired	186 21 42	74.7 8.4 16.9
**Family income adequacy for living expenses**		
Less than monthly expenses Equal to monthly expenses More than monthly expenses	135 84 30	54.2 33.7 12.1

Note. *N* = 249. Participants were on average 56.42 years old (SD = 5.97)

### Construct validity

A Kolmogorov-Smirnov test was used to assess data normality among 249 participants. Subsequently, the WSKAQ factor structure was identified using exploratory factor analysis (EFA) in SPSS software version 27 [32]. The Kaiser–Meyer–Olkin (KMO) and Bartlett’s tests for sphericity were employed to ascertain sampling adequacy and the suitability of the factor analysis. Principal component analysis with varimax rotation was then conducted to extract latent factors and their appropriate items. Items were allocated to a factor based on commonalities exceeding 0.3 [33].

### Discriminative validity (known group validity)

Known group validity (discriminative validity) was performed by the relation between education and self-care knowledge. Attitude is explained by three effects: The first effect is that low education is a risk factor for insufficient knowledge, and the negative attitude toward self-care because an individual with higher education had a better understanding of self-care and higher health literacy [34, 35]. The second level of education was shown to be an independent predictor of positive attitudes and degree of empowerment regarding self-care because of association with practices at the community level, such as attending meetings and educating other members of the family and/or neighbors [36, 37]. The selected menopausal women were divided into two groups based on education level (lower than diploma and equal to or higher than diploma). Then, self-care knowledge and attitude were compared between groups using an independent t-test.

### Reliability

Finally, the Internal consistency of the total WSKAQ was assessed using Cronbach’s α coefficient and McDonald’s Omega. Internal consistency of knowledge and attitude subscales was examined by Kuder-Richardson and Cronbach’s alpha, respectively [23, 38]. Kirk and Miller (1986) stated that the reliability of a questionnaire can be determined by measuring the correlation value between the scores of each item and the total score (item-total correlation) [39]. In addition, Cohen (1988) classified the values into three categories: small (0.10 to 0.29), medium (0.30 to 0.49), and high (0.50 to 1.00) [23]. Spearman’s rho test was used to evaluate the correlation value of each subscale score with other and total score [40].

## Results

### Participants

In this study, 249 menopausal women participated with a mean ± SD of age and menopause age of 56.42 ± 5.97 and 48.90 ± 4.76 years, ranging from 45 to 65 and 30 to 61 years, respectively. A large proportion of the samples were married (196; 78.7%), held an education < diploma (125; 50.2%), and were homemakers (186; 74.7%). Further, most samples reported a family income lower than their monthly expenses (135; 54.2%). Table 2 summarizes the collected demographic details.

### Construct validity

The construct validity section of the study began with the Kolmogorov–Smirnov test, which indicated a lack of items normality (*p* < 0.001). However, the results of Bartlett’s test of sphericity (χ2 = 83.924; *p* < 0.001) and the KMO test (KMO = 0.637) demonstrated sampling adequacy, justifying the use of factor analysis for seven-item self-care knowledge subscales and undergoing principal component analysis with varimax rotation. EFA identified two factors that accounted for 47.29% of the total data variance. A panel of health promotion experts named two factors using the component of self-care in a scientific paper [8–11] as a reference framework, including screenings (three items) and lifestyle (three items) (Table 3). In addition, item 1 was not allocated to a factor (commonalities < 0.3). The results of Bartlett’s test of sphericity (χ2 = 802.081; *p* < 0.001) and the KMO test (KMO = 0.878) demonstrated sampling adequacy, justifying the use of factor analysis for the seven-item self-care attitude subscale and undergoing principal component analysis with varimax rotation. EFA identified one factor that accounted for 55.50% of the total data variance (Table 3).

**Table 3:** Results from a factor analysis of the 14-items WSKAQ for Iranian menopausal women

7-Items of self- care knowledge Total variance: 47.29%	Factor loading		7-Items of self- care attitude Total variance: 55.50%	Factor loading
	Screenings	Life style		
Item 1	-0.16	0.28	Item 1	**0.79**
Item 2	0.26	**0.57**	Item 2	**0.81**
Item 3	0.07	**0.78**	Item 3	**0.89**
Item 4	**0.68**	0.04	Item 4	**0.82**
Item 5	**0.78**	0.26	Item 5	**0.71**
Item 6	**0.54**	0.25	Item 6	**0.50**
Item 7	0.12	**0.58**	Item 7	**0.58**

Note. *N* = 249. The extraction method was principal axis factoring with a varimax (with Kaiser Normalization) rotation. Factor loadings above 0.30 are in bold

**Fig. 2:**
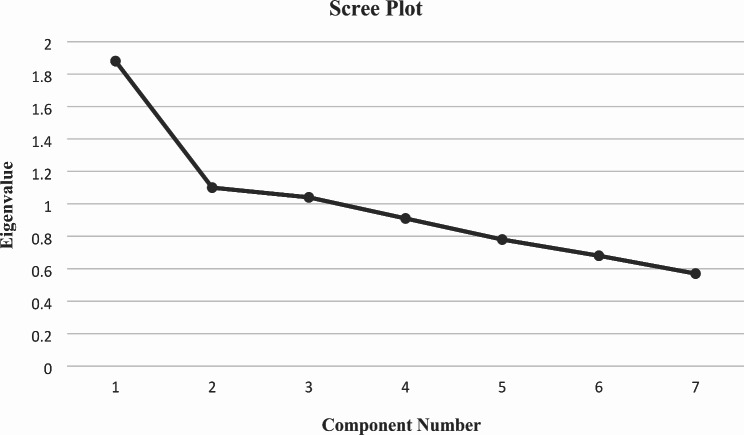
The scree plot of the factor analysis of the 7-items self- care knowledge for menopausal women

**Fig. 3:**
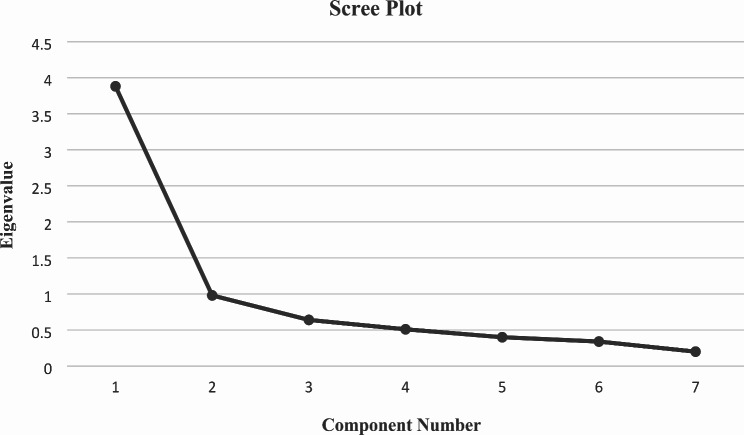
The scree plot of the factor analysis of the 7-items self- care attitude for menopausal women

Additionally, as shown in Figs. 2 and 3, the scree plot revealed that the selection of an eigenvalue greater than one led to the first significant change in the curve at factor two. Accordingly, the number of factors could range from one to three, as recommended by various sources [25].

### Discriminative validity (known group validity)

Independent t-test showed that menopausal women with education level equal to or higher than diploma have higher mean scores of self-care attitude than those with education level lower than diploma [t (247) = 2.72, Cohen’s d: 0.34, *p* = 0.007)] (Table 4).

**Table 4:** Self- care knowledge and attitude score comparison between groups

Subscales	< diploma		≥ diploma		t (df)	*p*	Cohen’s d
	Mean	SD	Mean	SD			
Self- care knowledge	4.76	1.39	4.63	1.17	0.79 (247)	0.42	0.10
Self- care attitude	29.48	3.65	30.70	3.47	2.72 (247)	0.007	0.34

Note. Mean parameter values for each of the analyses are shown for the education lower than diploma group (*n* = 125) and education equal to or higher than diploma group (*n* = 124), as well as the results of *t* tests (assuming unequal variance) comparing the parameter estimates between the two groups

### Reliability analysis

The Cronbach’s α coefficients and McDonald’s Omega values for the final questionnaire were 0.77 and 0.78, respectively. The subscale of self-care knowledge Kuder- Richardson coefficients were 0.79, and Cronbach’s α coefficients for self-care attitude was 0.81. Spearman’s rho test showed a solid correlation value of the self-care knowledge (r: 0.54) and attitude (r: 0.95) scores with the total score of questionnaires (*p* < 0.001), respectively. The correlation between the subscales was medium (r: 0.30, *p* < 0.001) (Table 5).

**Table 5:** The values of correlation and internal consistency and ICC of subscales

Variables	Correlation value of items to subscales		Internal consistency method	Value	ICC(95%CI)	Correlation Value of subscales Score with Total Score
Self- care knowledge	Item2 Item3 Item 4 Item 5 Item6 Item 7	r: 0.27^*^ r: 0.19^*^ r: 0.28^*^ r: 0.28^*^ r: 0.30^*^ r: 0.26^*^	Kuder- Richardson	0.79	0.79 (0.73–0.88)	r: 0.54^**^
Self- care attitude	Item 1 Item 2 Item 3 Item 4 Item 5 Item 6 Item 7	r: 0.63^*^ r: 0.69^*^ r: 0.76^*^ r: 0.65^*^ r: 0.58^*^ r: 0.42^*^ r: 0.48^*^	Cronbach’s alpha	0.81	0.81 (0.77–0.84)	r: 0.95^**^

Note.^*^p < 0.05.^**^p < 0.001.

Item-total (biserial) correlations revealed a significant correlation between each item and the total score of self-care knowledge. The biserial correlation values were between 0.19 and 0.30 (*p* < 0.05), where the minimum and maximum values belonged to the item about lifestyle and screenings, respectively. Based on Cohen’s (1988) classification [23], one item had a medium correlation, and five items had a small correlation with the total score (Table 5).

The biserial correlation values of self-care attitude were between 0.42 and 0.76 (*p* < 0.05). Based on Cohen’s (1988) classification [23], two items had a medium correlation, and five items had a high correlation with the total score (Table 5).

## Discussion

Self-care is essential for the well-being of people, communities, and the planet, and self-care knowledge and attitude are primary factors for self-care behaviors [41, 42]. Therefore, it is recommended to develop a standard questionnaire to assess the knowledge and attitude related to self-care [24]. However, insufficient studies have been conducted to evaluate the validity of self-care knowledge and attitudes [43], specifically for menopausal women. Therefore, this study aimed to evaluate the validity and reliability of the WSKAQ as a suitable and short tool for Iranian menopausal women because the main limitation of long questionnaires was the vagueness of their validity and reliability. In addition, the brevity of the questionnaire was highlighted as a beneficial attribute for research purposes, ensuring quicker and more precise completion [23, 24].

The results revealed that two components extracted from the self-care knowledge in the EFA explained 47.29% of the variance. The mentioned components included ‘lifestyle’ three items (fruit/vegetable consumption, physical activity definition, and definition of social support for decreased stress) and ‘screenings’ three items (periodic time of pap smear, breast self-examination, and visit to the gynecologist/midwife). In addition, one component extracted from the self-care attitude in the EFA explained 55.50% of the variance, including seven items (Table 1). Contrastingly, the self-care activities in scientific papers are fruit/vegetable consumption, physical activity, and regular preventive care such as screenings and stress control [8–11].

The present study revealed that higher education is associated with a more positive self-care attitude. Similarly, this relationship has been confirmed in other studies [36, 37]. Therefore, family support and education regarding self-care by healthcare providers were key influencers to self-care attitudes in individuals with low levels of education [44].

In the present study, all three types of reliability were examined, and the current questionnaire had desirable reliability. In addition, the correlation between self-care knowledge and attitude was medium. However, a lack of studies evaluating the validity properties of self-care knowledge and attitude is apparent [43], requiring further research.

### Strengths and limitations

The strengths of this study were manifold, and it successfully validated the WSKAQ specifically for menopausal women. Another significant strength was that the study rigorously assessed validity using EFA and known-group validation. Reliability was comprehensively evaluated using all three types of reliability.

However, the study also had limitations. One primary limitation was conducting the study in urban health centers, focusing solely on urban women. This geographic and demographic limitation suggests the need for future research in rural health centers and with rural women. Another limitation is the educational level of the participants; only women with at least primary education were included, limiting the generalizability of the findings to illiterate women. Additionally, the reliance on self-reported data introduced potential biases. Self-report studies are susceptible to errors and can be affected by participants’ emotional states when completing the questionnaires.

## Conclusion

Based on the results, the 13-item version of the WSKAQ in the Iranian context showed acceptable validation properties and reliability, making it practical for measuring self-care knowledge and attitude in menopausal women. Furthermore, the importance of self-care knowledge and attitude in women’s health underscores the need for an accurate evaluation tool. This paper aimed to facilitate more comprehensive and insightful cross-cultural self-care knowledge and attitude studies.

## Data Availability

The datasets used and/or analyzed during the current study are available from the corresponding author upon reasonable request.
